# Factors related to survival outcomes following orbital exenteration: a retrospective, comparative, case series

**DOI:** 10.1186/s12886-018-0850-y

**Published:** 2018-07-28

**Authors:** Orapan Aryasit, Passorn Preechawai, Chakree Hirunpat, Orasa Horatanaruang, Penny Singha

**Affiliations:** Department of Ophthalmology, Faculty of Medicine, Prince of Songkla University, 15, Kanjanavanich Rd, Kohong, Hat Yai, Songkhla, 90110 Thailand

**Keywords:** Orbital exenteration, Predictive factor, Metastasis, Overall survival

## Abstract

**Background:**

Orbital exenteration is a disfiguring procedure that aims to achieve local control. It is commonly a part of the management of malignant orbital tumor which is a life-threatening condition. It is necessary to determine predictive factors associated with overall survival (OS) following orbital exenteration.

**Methods:**

This was a retrospective, comparative, case series of 39 patients with malignant tumors who underwent orbital exenteration. Patient records were reviewed for age, clinical presentation, preoperative visual acuity (VA), tumor size, surgical margin, tumor invasiveness, recurrent disease, and status of distant metastasis. Kaplan-Meier curves were used to assess OS and event-free survival (EFS). The predictive factors related to OS were identified using multivariate analysis.

**Results:**

The mean age was 62.9 years (range, 5.5 to 89.7 years), 68.4% presented with VA < 20/400. The mean size of all tumors was 32 ± 18 mm. Distant metastasis at diagnosis was reported in 11 patients (28.2%). Twenty-two patients died during follow-up. The median OS and EFS were 3.89 years and 3.01 years, respectively. The predictive factors for worse OS on multivariate analysis were preoperative VA < 20/400 (adjusted hazard ratio [aHR] 4.67, *P* = 0.003), tumor size larger than 20 mm (aHR 3.14, *P* = 0.022,) and positive distant metastasis at diagnosis (aHR 15.31, *P* <  0.001).

**Conclusions:**

The prognostic factors for poor survival outcome following orbital exenteration were a preoperative VA < 20/400, tumor size > 20 mm, and distant metastasis at diagnosis mostly due to patient negligence.

## Background

Orbital exenteration is a disfiguring procedure which removes all of the orbital contents including the periosteum and eyelids with or without the orbital bone. Modern exenteration was first described by George Bartisch in 1583 (cited by Goldberg et al. [1]). A number of diseases require orbital exenteration to achieve local control; for example, destructive tumors that have spread to the orbit, lacrimal gland malignancies, and fungal infections. About 50% of exenteration cases originate from the eyelids or periocular skin [1, 2].

Most publications on predictive factors associated with survival outcomes following orbital exenteration were reported from developed countries. Wong et al. reported that survival was significantly more closely related to the histopathological diagnosis (mostly basal cell carcinoma) than surgical margins [3]. Otherwise, positive final surgical margin had a poor prognosis in patients who underwent orbital exenteration for advanced periorbital skin cancer [4]. In addition, bone erosion and perineural invasion were the predictive factors for poor survival of orbital exenteration [4, 5].

The goal of our study was to examine the predictive factors related to overall survival (OS) following orbital exenteration in patients with malignancy. We considered the following factors that could predict survival: age, presenting symptoms and their duration, preoperative visual acuity (VA), tumor size (greatest dimension of the lesion in millimeters), histopathological diagnosis, surgical margin, tumor invasiveness (lymphovascular, perineural, bony), recurrent disease, and status of metastasis.

## Methods

### Study design

This retrospective study included patients admitted for total or extended orbital exenteration performed between January 2006 and February 2016 at Songklanagarind Hospital which is a major tertiary-care center and university hospital in southern Thailand. Approval was obtained from the Ethics Committee of the Faculty of Medicine, Prince of Songkla University, and this study adhered to the tenets of the Declaration of Helsinki. We excluded patients with non-malignancy who underwent orbital exenteration.

### Data collection

Patient records were reviewed for age, gender, presenting symptoms and their duration, preoperative VA after referral from a primary or secondary care center, indication for surgery, tumor origin, tumor size, histopathological diagnosis, surgical margin, tumor invasiveness (lymphovascular, perineural, bony), status of distant metastasis, surgical complications, recurrent disease, date of death (if applicable), and cause of death of deceased patients. We defined a large tumor as > 20 mm in greatest dimension. Poor preoperative VA was defined as < 20/400 using the WHO blindness classification [6] as a potential predictive factor affecting OS.

### Statistical analysis

Data were analyzed using Stata Statistical Software (STATA MP 14.1. StataCorp LP). Event-free survival (EFS) was measured from the date of orbital exenteration to recurrent disease or death due to any cause. OS was defined as the date of orbital exenteration until last follow-up or death. Patients without an event or death were censored at the time of last known follow-up or May 1, 2017. VA loss, large tumor, extraocular muscle involvement, tumor invasiveness, unclear surgical margin, recurrent disease, and distant metastasis at diagnosis were the factors used in the Kaplan Meier analysis with a confidence interval [7]. The log-rank test was used to potentially predict a poor prognosis for OS. The Mann-Whitney U test was also used for the statistical analysis.

Multivariate models were constructed including minimal sets of adjustment variables indicated by a directed acyclic graph using DAGitty Version 3.0 (Johannes Textor, Utrecht University, The Netherlands) to minimize bias in the estimation. The causal diagram between the variables of interest and covariables was created based on causal assumptions, and the total effect of influencing survival was reported. Cox proportional hazards models were used to analyze the predictive factors for survival outcomes following orbital exenteration. A *P* value < 0.05 was considered to indicate statistical significance.

## Results

### Patient data and histopathologic diagnosis

From a total of 41 patients who underwent orbital exenteration over the study period, only 2 patients were excluded: 1 invasive aspergillosis and 1 mucormycosis. Thirty-nine patients (21 males, 18 females) were enrolled in the study with a mean age of 62.9 ± 20.4 years (range, 5.5 to 89.7 years). Fourteen different tumors were identified (Table 1). The most common tumor origins in the exenterated patients were the conjunctiva and the eyelids. In addition, 14 patients (9 males and 5 females) with a mean age of 67.1 years were diagnosed with squamous cell carcinoma. The most common presenting symptoms and signs were restriction of extraocular movement (74.4%), followed by blurry vision (68.4%), mass (66.7%), and eye pain (41.0%). Eleven patients with distant metastasis had blurry vision (81.8%) and eye pain (45.5%).

**Table 1:** Tissue origin and histological diagnosis of 39 exenterated cases

Origin	Histological diagnosis	Number of cases
Conjunctiva	Squamous cell carcinoma	11
	Malignant melanoma	1
	Mucoepidermoid carcinoma	1
Eyelid	Squamous cell carcinoma	3
	Sebaceous cell carcinoma	3
	Basal cell carcinoma	2
	Malignant melanoma	2
	Adenocarcinoma	2
Lacrimal gland	Adenoid cystic carcinoma	5
	Adenocarcinoma	1
Globe	Choroidal melanoma	2
	Retinoblastoma	2
Orbit	Malignant fibrous histiocytoma	3
	Apocrine carcinoma	1

The preoperative VA was equal to or better than 20/400 in 12, less than 20/400 to light perception in 13, no light perception in 13, and no data available in 1 case. The mean tumor size was 32 ± 18 mm (range, 10 to 100 mm), and mean duration of presenting symptoms was 67.9 weeks (range, 4.3 weeks to 6.0 years). Five patients had regional nodal metastasis at initial diagnosis. Distant metastases were detected in 11 patients (liver in 3, brain in 2, lung in 2, bone in 2, and multiple sites in 2) that consisted of 3 squamous cell carcinomas, 3 malignant melanomas, 3 adenoid cystic carcinomas, 1 retinoblastoma, and 1 apocrine carcinoma. The median sizes of tumor in the non-metastasis group versus metastasis group were 27.5 mm and 35 mm, respectively, but the difference between the groups was not statistically significant (*P* = 0.332). Of the 39 patients, 32 patients had neglected their disease, 4 had delayed initial diagnosis, and only 3 underwent primary treatment before orbital exenteration.

### Treatment modalities and outcome

Of the 39 patients, 31 underwent total orbital exenteration and 8 underwent extended orbital exenteration. Six patients underwent additional resection: 2 parotidectomies, 1 neck node dissection, 1 craniofacial surgery, 1 maxillectomy, 1 ethmoidectomy, and 1 lateral rhinectomy. The orbital reconstructions involved 23 skin grafts, 12 bare bones, and 4 local flaps. Ten patients received only postoperative radiation, 7 received combined chemoradiation, and 1 received only chemotherapy.

**Fig. 1:**
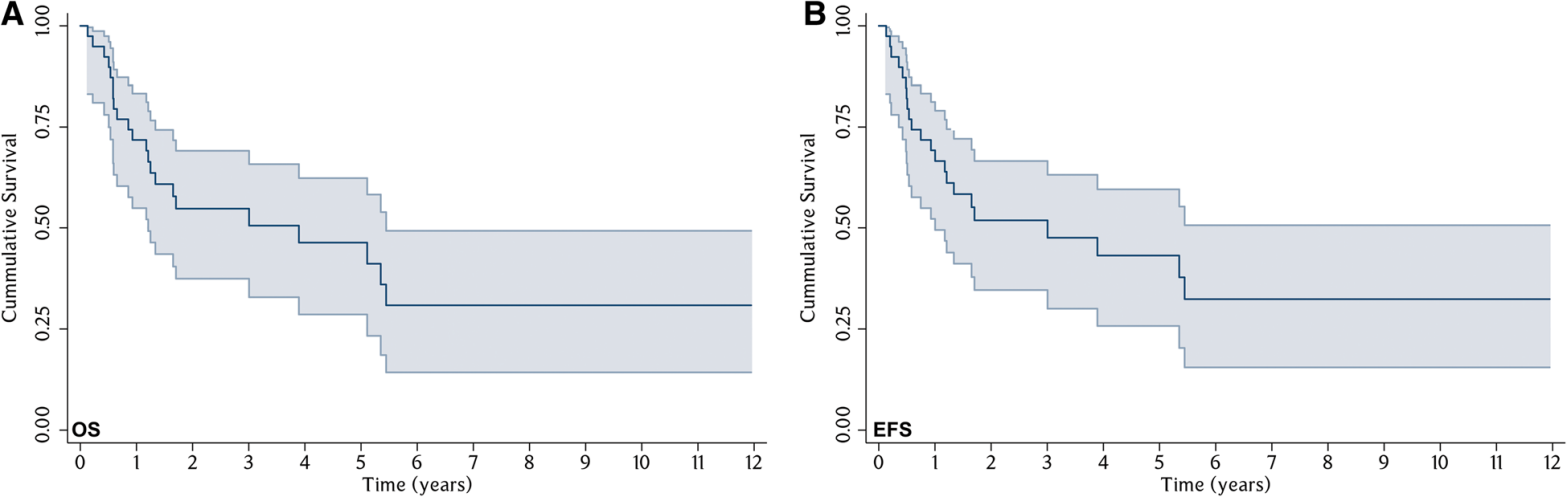
Kaplan-Meier survival analysis for 39 cases: (**a**) Overall survival (OS). (**b**) Event-free survival (EFS). The shaded areas represent 95% confidence interval (CI)

Seven patients experienced recurrence at the mean time of 34.7 weeks (range, 10.4 weeks to 1.38 years) with a mean follow-up time of 3.1 years (range, 1.6 months to 12.0 years). Seventeen patients were living and 22 had died. Deaths in 3 patients were unrelated to the tumor. The median OS and EFS of all exenterated patients were 3.89 years and 3.01 years, respectively. The Kaplan-Meier estimates for OS at 1, 3, and 5 years were 69.1%, 50.5%, and 41.1%, respectively (Fig. 1a). The EFS at 1, 3, and 5 years were 66.6%, 47.5%, and 37.8%, respectively (Fig. 1b).

The log-rank test was used to identify variables significantly associated with OS (Table 2). The prognostic factors that could significantly predict inferior survival outcome in our study using univariate analysis were preoperative VA < 20/400 (*P* = 0.018), tumor size > 20 mm (*P* = 0.032), distant metastasis at diagnosis (*P* <  0.001), and recurrent disease (*P* = 0.043).

**Table 2:** Univariate analysis for various factors related to overall survival (OS)

Characteristic	survival					*P* value
	n/N	Median survival time (years)	1 year (%)	3 years (%)	5 years (%)	
Overall rate	22/39	3.89	69.1	50.5	41.2	
Gender						
Male	11/21	5.11	71.4	60.3	46.5	0.269
Female	11/18	1.34	66.2	19.3	19.3	
Mean age (years)						
≤ 63	11/18	1.65	55.0	40.1	30.0	0.516
> 63	11/21	5.35	76.2	51.9	41.5	
Mean duration of presenting symptom (weeks)						
≤ 67.9	16/26	3.89	65.2	51.8	41.5	0.967
> 67.9	6/13	1.70	69.2	48.5	48.5	
Restriction of extraocular movements						
No	4/10	5.35	90.0	75.0	56.0	0.093
Yes	18/29	1.65	58.0	36.2	29.0	
Mass						
No	6/13	5.35	69.2	29.7	29.7	0.965
Yes	16/26	3.01	69.2	49.0	37.8	
Eye pain on presentation						
No	13/23	3.01	69.6	55.8	42.5	0.646
Yes	9/16	3.89	68.8	40.1	20.1	
VA						
≥ 20/400	3/12	10.1	91.7	68.8	68.8	0.018*
< 20/400	18/26	1.65	57.4	38.1	30.5	
Tumor size						
≤ 20 mm	4/13	11.95	76.2	67.7	67.7	0.032*
> 20 mm	18/26	1.65	65.4	41.4	27.6	
Histopathologic diagnosis						
Non-squamous cell carcinoma	15/25	3.01	63.8	43.8	37.6	0.528
Squamous cell carcinoma	7/14	5.35	78.6	50.3	25.1	
Tumor origin						
Lid	6/12	3.01	83.3	44.4	22.2	0.660
Lacrimal, globe, and orbit	8/14	0.85	50.0	40.0	40.0	
Conjunctiva	8/13	5.11	69.2	52.8	39.6	
Surgical margins						
Unclear margin	7/14	5.45	71.4	52.1	39.1	0.597
Clear margin	15/25	3.89	68.0	44.1	35.3	
Tumor invasiveness (lymphovascular, perineural, bony)						
No	15/29	3.89	65.5	48.6	41.7	0.453
Yes	7/10	3.01	80.0	40.0	20.0	
Status of metastasis						
No distant metastasis	9/23	5.45	91.3	70.6	62.8	< 0.001*
Regional nodal metastasis	3/5	3.01	60.0	60.0	0.0	
Distant metastasis	10/11	0.59	24.2	0.0	0.0	
Adding resection						
No	18/33	3.89	63.5	47.9	41.9	0.815
Yes	4/6	3.01	83.3	33.3	0.0	
Radiation						
No	12/22	3.89	72.7	52.0	36.4	0.579
Yes	10/17	5.11	64.7	37.8	37.8	
Chemotherapy						
No	16/31	3.89	77.3	53.5	41.3	0.197
Yes	6/8	0.65	37.5	18.8	18.8	
Recurrent disease						
No	16/32	5.35	74.9	55.5	44.1	0.043*
Yes	6/7	0.85	28.6	0.0	0.0	

*n* deceased patients*, N* total patients*, VA* Visual acuity, *P value < 0.05

A clear surgical margin was obtained in 25 cases (64.1%). Of these, 4 cases had regional nodal metastasis and 6 had distant metastasis at initial diagnosis. Fourteen patients were reported to have an unclear surgical margin (6/6 lacrimal gland, 3/4 orbit, 3/13 conjunctiva, 2/12 eyelid). The OS rates for clear surgical margins at 1 and 5 years were 68.0% and 35.3%, respectively. We also analyzed the OS for unclear surgical margins at 1 and 5 years and the results were 71.4% and 39.1%, respectively.

**Fig. 2:**
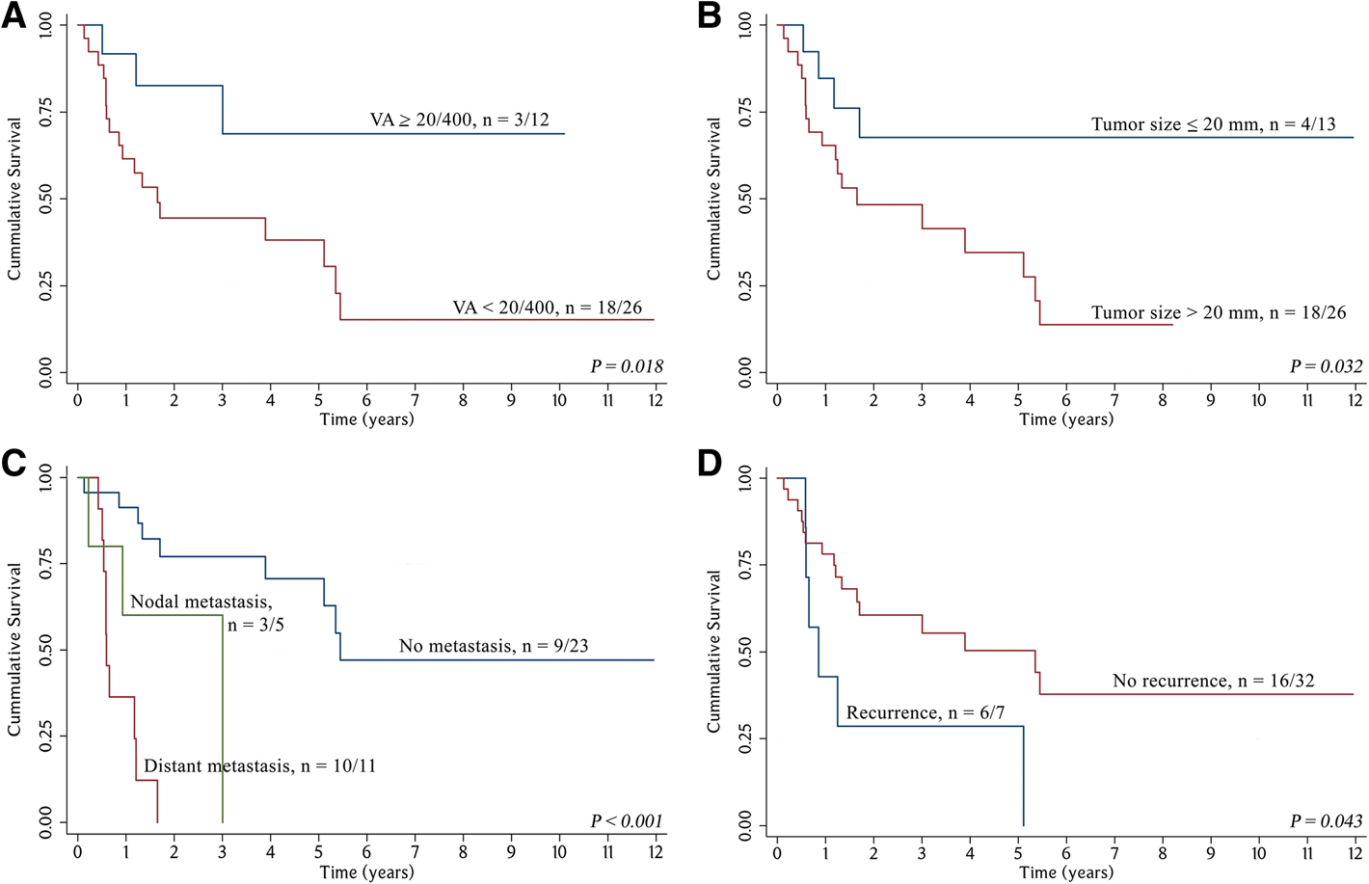
Kaplan-Meier survival analysis for each group: (**a**) Overall survival (OS) for patients with VA ≥20/400 versus VA < 20/400 (**b**) OS for patients with tumor size ≤20 mm versus tumor size > 20 mm. (**c**) OS for patients with no distant metastasis, regional nodal metastasis versus distant metastasis. (**d**) OS for patients with or without recurrent disease

The 1-year and 5-year OS rates were 91.7% and 68.8%, respectively, for patients with preoperative VA ≥ 20/400 which were superior compared with VA < 20/400 (Fig. 2a). The Kaplan-Meier estimates for OS for tumor size > 20 mm at 1 and 5 years were 65.4% and 27.6%, respectively, whereas for tumor size ≤ 20 mm at 1 and 5 years, the OS estimates were 76.2% and 67.7%, respectively (Fig. 2b). Patients with distant metastasis at diagnosis had an inferior OS (24.2% and 0% at 1 and 5 years, respectively) in comparison with patients without distant metastasis (91.3% and 62.8% at 1 year and 5 years, respectively) (Fig. 2c). Recurrent disease was significantly associated with worse OS (Fig. 2d).

Multivariate analysis of OS revealed three predictive factors that were independently related to survival outcome following orbital exenteration: preoperative VA < 20/400 (adjusted hazard ratio [aHR] 4.67, 95% confidence interval [CI] 1.27 to 17.12, *P* = 0.003), tumor size > 20 mm (aHR 3.14, 95% CI 1.06 to 9.32, *P* = 0.022), and positive distant metastasis at diagnosis (aHR 15.31, 95% CI 4.25 to 55.19, *P* <  0.001) (Table 3).

**Table 3:** Multivariate analysis of predictors associated with overall survival (OS) showing total effect

Variable	Minimally sufficient adjustment set	Exposure variable: Level	Adjusted Hazard Ratio (95% CI)	*P* value
Mean age (years)		≤ 63	1	
		> 63	0.75 (0.33, 1.75)	0.519
Mean duration of presenting symptom (weeks)	- Histopathologic diagnosis	≤ 67.9	1	
		> 67.9	1.19 (0.41, 3.45)	0.751
Restriction of extraocular movements	- Mean duration of presenting symptom	No	1	
	- Tumor invasiveness	Yes	2.39 (0.71, 7.98)	0.199
	- Tumor origin			
Mass		No	1	
		Yes	1.02 (0.40, 2.64)	0.965
Eye pain on presentation	- Histopathologic diagnosis	No	1	
		Yes	1.40 (0.56, 3.48)	0.476
VA	- Mean age	≥ 20/400	1	
	- Histopathologic diagnosis			
		< 20/400	4.67 (1.27, 17.12)	0.003*
	- Tumor origin			
	- Tumor size			
Tumor size	- Mean duration of presenting symptom	≤ 20 mm	1	
	- Histopathologic diagnosis	> 20 mm	3.14 (1.06, 9.32)	0.022*
Histopathologic diagnosis	- Mean age	Non-squamous cell	1	
	- Tumor origin	Squamous cell	0.58 (0.15, 2.30)	0.435
Tumor origin		Lid	1	
		Lacrimal, globe, and orbit	1.57 (0.54, 4.59)	0.674
		Conjunctiva	1.09 (0.37, 3.16)	
		Conjunctiva	1	
		Lacrimal, globe, and orbit	1.44 (0.53, 3.91)	
Surgical margins		Clear margin	1	
		Unclear margin	0.78 (0.32, 1.94)	0.593
Tumor invasiveness (lymphovascular, perineural, bony)		No	1	
		Yes	1.41 (0.57, 3.48)	0.466
Status of metastasis	- Mean duration of presenting symptom	Negative	1	
	- Histopathologic diagnosis	Regional nodal metastasis	3.32 (0.76, 14.52)	< 0.001*
	- Tumor size	Distant metastasis	15.31 (4.25, 55.19)	
Adding resection	- Tumor invasiveness	No	1	
	- Recurrent disease			
		Yes	0.57 (0.15, 2.23)	0.403
	- Surgical margins			
Radiation	- Status of metastasis	No	1	
	- Surgical margins			
		Yes	1.27 (0.45, 3.56)	0.654
Chemotherapy	- Status of metastasis	No	1	
		Yes	1.76 (0.63, 4.93)	0.298
Recurrent disease		No	1	
		Yes	2.62 (0.99, 6.91)	0.070

*CI* confidence interval*, LR* likelihood ratio, *VA* Visual acuity, *P value < 0.05

The OS rates of 14 patients with squamous cell carcinoma at 1, 3, and 5 years were 78.6%, 50.3%, and 25.1%, respectively. Distant metastasis at initial diagnosis and recurrent disease were significantly associated with worse OS of squamous cell carcinoma patients using the log-rank test (*P* = 0.001 and *P* = 0.020, respectively).

## Discussion

This 10-year study found malignancies in 39 of 41 patients (95.1%) who underwent an orbital exenteration. These results were similar to previously published data [8–11]. Before 1990, the most common histopathologic diagnosis in patients requiring orbital exenteration was basal cell carcinoma (23.1% to 35.5%) [12–14]. Of note, an increasing trend was reported in the cases of squamous cell carcinoma due to globe or periorbital invasion that could not be managed by a simple surgical excision which comprised 32.3% to 38.4% of all exenterated patients [10, 15, 16].

Our study also reported the most common indication for orbital exenteration was squamous cell carcinoma in 34.1% (conjunctiva in 11 and eyelid in 3). We found a low proportion of basal cell carcinoma (5.1%). Ali et al. reported that ocular surface squamous neoplasia (44.4%) and sebaceous gland carcinoma (18.5%) were the most common indications for orbital exenteration in India [17]. Ocular surface squamous neoplasia is predominant in Asian and African countries because of chronic sun exposure and agricultural occupations [18, 19]. Of the 14 squamous cell carcinoma patients in our report, 64.3% were males. Demographic data of the current study also suggested a male predominance and the mean age of nearly 70 years in those patients with squamous cell carcinoma corresponded to the findings of prior studies [20, 21]. Our study revealed a low rate of basal cell carcinoma in association with orbital exenteration. This might be due to the fact that basal cell carcinoma is a less aggressive tumor and the patient usually has time to recognize it.

Interestingly, we revealed that tumor-related survival was significantly better for patients with a preoperative VA ≥20/400 than for patients with a VA < 20/400. In addition, the common clinical presentation in our study was blurry vision which presented in nearly 70%. Therefore, preoperative VA was one of the predictive factors related to OS. A possible reason for this is the origin of the tumor (i.e. the globe). For example, retinoblastoma and choroidal melanoma, which invade the sclera and involve the optic nerve or the orbit, are associated with a low rate of survival in spite of orbital exenteration [22–25]. A second reason is that malignant tumors of periorbital structures that affect the vision are typically invasive and highly aggressive in behavior. Even though the patient may have mild visual impairment in case of partial globe or optic nerve involvement, we recommend performing an orbital exenteration before the tumor advances further.

Although we were able to achieve local control with clear surgical margins in 25 patients (64.1%), 10 of these patients had positive metastasis at initial diagnosis (4 patients had regional nodal metastasis [2 squamous cell carcinomas and 2 malignant melanomas] and 6 had distant metastasis [3 malignant melanomas, 2 squamous cell carcinomas, and 1 retinoblastoma]). Notably, we confirmed that the clear surgical margin group had a high rate of nodal or distant metastasis at diagnosis (40%). The log-rank test revealed that clear versus unclear surgical margins did not show statistical significance in terms of OS (*P* = 0.597). Since a high proportion of metastasis was found in patients with clear surgical margins, we propose that micrometastasis occurred prior to orbital exenteration. However, surgical margins play an important role in controlling the site of the malignant tumors. Mouriaux et al. also reported that surgical margins significantly affected the control of local recurrence, which was not related to survival [15]. On the other hand, Gerring et al. reported that an unclear surgical margin was the only factor associated with a poor prognosis on multivariate analysis in patients undergoing orbital exenteration due to advanced periorbital non-melanoma skin cancer [4]. For the unclear surgical margin cases, adjuvant radiotherapy or chemotherapy or both increased the surgical cure rate [9, 10, 26]. Tumor invasiveness (lymphovascular, perineural, bony) was also not associated with a poor survival outcome which was contrary to the findings of previous studies [8, 27]. However, it is important to recognize that our study had too few cases to detect statistical significance.

In this current study, tumor size greater than 20 mm at presentation was considered a large tumor and this significantly affected OS. Several studies of eye cancer also reported that larger tumor size posed a risk of local recurrence or metastasis which was possibly associated with the inferior survival outcome [28, 29]. Therefore, patient awareness and education is important to detect the disease in the early stage and receive proper management.

Distant metastasis at diagnosis is defined following the tumor-node-metastasis staging system and is associated with a poor overall prognosis. Therefore, distant metastasis was a predictive factor that helped estimate survival in our study, in which 11 of the 39 orbital exenterations had distant metastasis at diagnosis. The OS rates of the metastasis group were 81.8% and 24.2% at 6 months and 1 year, respectively. All patients with distant metastasis at diagnosis died within 1.65 years. The design of our study did not compare the OS between the patients who received only palliative chemoradiation without an orbital exenteration and the patients who underwent an orbital exenteration and adjuvant chemoradiation. Although orbital exenteration has the surgical goal of complete excision, it can improve local control to eliminate aggressive growth in the cases of a very large tumor and eye pain. However, in the metastasis group, the surgeon should discuss the choices of reconstruction and probably prefer a more conservative surgery.

The 5-year OS rate of all patients in our study was 41.2%. This was lower compared to those of other studies because most of our patients in our study presented with large squamous cell carcinomas which pointed to patient negligence in treating the problem [2, 8, 30].

One prior study revealed the OS rate of non-basal cell carcinoma (mostly, squamous cell carcinoma) at 5 years was 58% [3]. However, in the advanced age group with periorbital squamous cell carcinoma, the 1-year OS rate was only 50.5% [21]. The current study reported the OS rate of squamous cell carcinoma patients at 5 years which was also low (25.1%). The possibility of death due to the histopathologic diagnosis was higher among squamous cell carcinoma patients who had distant metastasis at diagnosis or the recurrent disease or both.

A limitation of our study was its retrospective design. However, orbital exenteration is a rare procedure. During the study period, we performed only 4 cases per year in our institution. Therefore, conducting a prospective study would be difficult. Additionally, the short follow-up time limited our ability to identify the real number of cases with a local recurrence**.** The follow-up time needed is more than 3 years. Furthermore, this study included various histological types of tumors. Although, our study reported the OS of squamous cell carcinoma patients and two predictors that were significantly associated with worse prognosis, we were not able to classify the predictive factors affecting survival outcome for each histological type due to the small number of patients. The strength of this study is the information that it provides on predictive factors related to survival outcome of exenterated patients who have advanced tumors.

## Conclusions

In summary, orbital exenteration can control local malignant tumors, but it is questionable in the treatment of cases with distant metastasis. The most common indication in our study was squamous cell carcinoma of the conjunctiva. Poor preoperative VA, larger tumor size, and distant metastasis were significantly associated with worse OS. Although a clear surgical margin was not related to survival, we recommend performing a clear surgical margin procedure and treating the early-stage disease, including cases with a small tumor size. The information on potential factors to predict OS may support patient counseling and the most effective treatment modality.

## Abbreviations

aHR: adjusted hazard ratio
CI: confidence interval
DAG: directed acyclic graph
EFS: event-free survival
LR: likelihood ratio
OS: overall survival
SD: standard deviation
VA: visual acuity
